# Perceptions and attitudes of dental practitioners towards impacts of Covid 19 pandemic on clinical dentistry: a cross-sectional study

**DOI:** 10.1186/s12903-022-02457-y

**Published:** 2022-09-22

**Authors:** Xiaoyan Zhou, Jinlong Gao, Alexander C. L. Holden, Shanika Nanayakkara

**Affiliations:** 1 School of Dentistry, Faculty of Medicine and Health, The University of Sydney, Sydney, NSW Australia; 2 Sydney Dental Hospital, Sydney Local Health District, Surry Hills, NSW Australia

**Keywords:** COVID-19 pandemic, Dentistry, Dental practitioner, Infection control, Tele-dentistry, Patient care

## Abstract

**Background:**

The COVID-19 pandemic challenged all healthcare providers including dental practitioners. This cross-sectional study aimed to investigate the dental practitioners’ perceptions and attitudes towards the impacts of COVID-19 on their professional practice, career decision and patient care.

**Methods:**

Data was collected from dental practitioners registered in New South Wales (NSW), Australia using an online survey.

**Results and conclusion:**

Responses received from 206 dental practitioners revealed their perceptions and attitudes towards COVID-19 infection risk, clinical guidelines, and measures adopted to deliver patient care. Majority of participants perceived the risk of infection in dentistry was higher compared with other health professionals. Most dental practices have followed guidelines received from professional associations and adopted multiple measures such as providing hand sanitizer, social distancing, and risk screen, to ensure safe delivery of oral health care. Over 80% of dental practitioners raised concerns on patients’ accessibility to dental care during the pandemic. Despite tele-dentistry was introduced, almost half of the participants did not recognize tele-dentistry as an effective alternative. Moreover, negative impacts of COVID-19 pandemic on dental practitioner’s professional career have been reported, including lower practice safety, reduction in working hours and income. Noteworthy, one quarter of participants even considered changing their practice environment, moving sectors or even leaving their career in dentistry. However, majority of the dental practitioners are willing to stay in their current practice environment and continue their career in dentistry. Our observations demonstrate the systematic disruption to dental practice faced in Australia due to the COVID-19 pandemic. Providing dental practitioners with timely educational training and support is important to minimise negative impacts of the challenges and to optimise dental care.

**Supplementary Information:**

The online version contains supplementary material available at 10.1186/s12903-022-02457-y.

## Background

It has been over two years since the World Health Organization (WHO) declared COVID-19 as a global pandemic on 11 March 2020 [1], and the COVID-19 pandemic declaration is ongoing. Since then, all facets of life and work have been significantly disrupted. According to the updated statistics data updated on 25th April 2022, there have been approximately 512 million people infected with the novel Severe Acute Respiratory Syndrome Coronavirus 2 (SARS-Cov-2) and about 6.23 million death cases worldwide, with 5.85 million positive cases and 7,164 death in Australia [2]. COVID-19 is a respiratory illness caused by SARS-Cov-2, which can be transmitted through inhalation of respiratory droplets and aerosol particles or touching surfaces or objects where the virus can stay viable for prolonged period [3].

Front-line health-care workers are at high-risk of being infected with COVID-19 [4]. WHO estimates that over 100, 000 health and care workers may have died from COVID-19 in the period between January 2020 to May 2021 [5]. Dental practitioners are not exempt from the risk of being infected, considering common dental procedures generate aerosols, and dental practitioners are routinely exposed to potentially infectious body fluids, including saliva, blood and respiratory excretions.

**Fig. 1:**
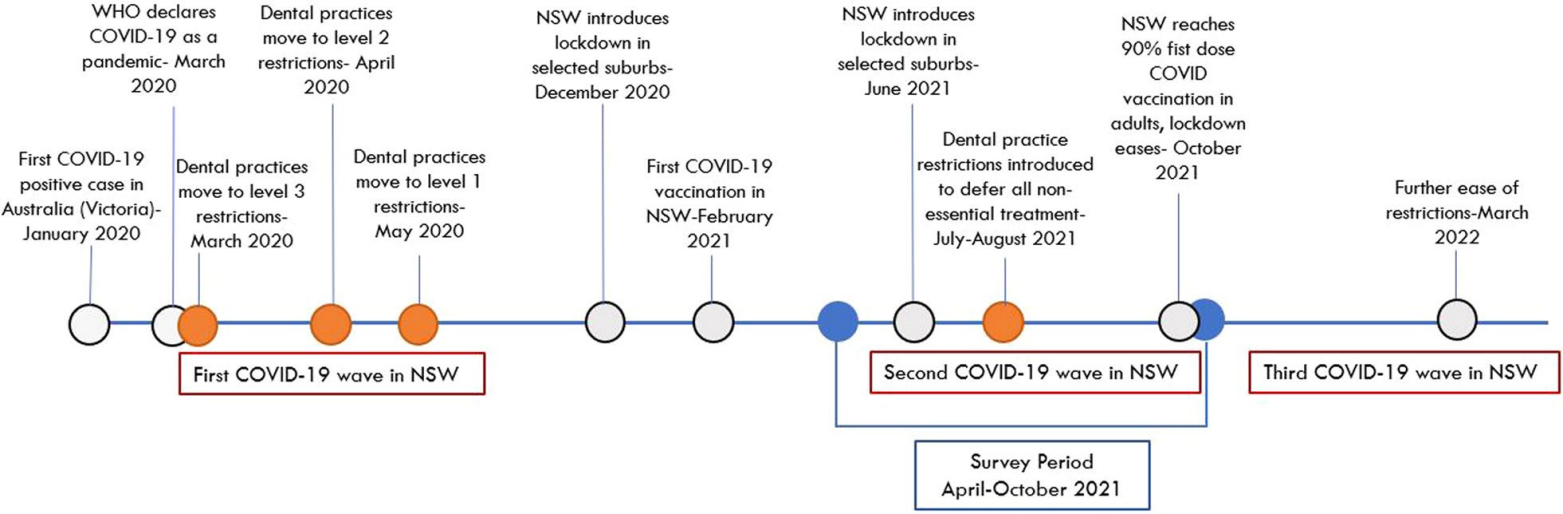
Key milestones of COVID 19 pandemic in New South Wales, Australia and survey period

To contain the spread of COVID-19, many countries and regions have adopted a range of policies and measures, including various degrees of restrictions and lockdowns. Since the first COVID-19 case was reported in January 2020 in Australia, case numbers have increased rapidly particularly in New South Wales (NSW) and Victoria. Since March 2020, the NSW state government introduced significant restrictions on movement and public gatherings. As indicated in Fig. 1, severity of the outbreak and the responses from the government and professional bodies have changed over the time [6–8]. Attitudes, perceptions and behaviours of the public and health care workers towards the pandemic and the restrictions have changed over different periods of the pandemic [9, 10].

Recognising the potential risk of infection to dental staff and cross infection between patients, the Federal Branch of the Australian Dental Association (ADA Inc) introduced different levels of restrictions and guidelines to dental practices to reduce disease transmission risks [11]. Professional associations like ADA Inc. developed publicly accessible resources to prepare dental practitioners with knowledge and awareness to mitigate infection risk when providing clinical patient care. Studies from different geographic locations have demonstrated dental practitioners have adequate knowledge and preparedness to identify COVID-19 symptoms, recognise modes of transmission and apply preventive measures [12–16]. However, limited research specifically examines the dental practitioners’ perceptions and attitudes towards the impacts of COVID-19 on their professional practice, career decision and patient care. Therefore, this cross-sectional study aimed to understand the attitudes and responses of dental practitioners in NSW during the COVID-19 pandemic and the impact of the pandemic on their clinical practice and career.

## Methods

This study was approved by the University of Sydney Human Research Ethics Committee (2020/712) and the study was performed in accordance with the Declaration of Helsinki.

### Study design and population

A cross-sectional study was conducted using an online survey in collaboration with the Dental Council of New South Wales (DCNSW) and the NSW Branch of the Australian Dental Association (ADANSW). The target population was dental practitioners (dentists, Oral Health Therapists, Dental Hygienists, Dental Therapists and Prosthetists) registered in NSW, Australia. In March 2021 there were 7394 registered dental practitioners NSW [17]. We expected to receive responses from 366 participants for this survey (5% margin of error, 95% confidence interval). Participation in this survey was voluntary and all registered dental practitioners in NSW were eligible to participate.

### Instrument development

An online survey was developed on the Qualtrics platform (Qualtrics, Provo, Utah) to explore the views and perceptions of dental practitioners on the impact of COVID-19 pandemic in their professional practice. The survey questionnaire was developed based on the available evidence and further refined following a review by an expert panel (academics and members from the DCNSW). Before distribution, the survey questions were pilot tested for validity with a group of dental practitioners (n = 5) using the retrospective think aloud protocol [18]. There were two sections in the finalised survey questionnaire, including 26 questions in the first section to collect data on the dental practitioners’ views and perceptions on the impact of COVID-19 pandemic on their dental practice and seven questions in the second section to collect demographic information (Additional file 1). Likert scale questions were used to measure the perceptions and attitude of the participants.

### Survey distribution and data collection

A link to the survey questionnaire was distributed to the dental practitioners registered in NSW through the DCNSW and the ADANSW. The survey was open from 1^st^ April to 31^st^ October 2021. Submission of the completed survey was considered as informed consent to participate in the study.

### Data analysis

Survey responses were downloaded from Qualtrics Survey Software and analyzed using IBM SPSS statistics software (Version 26, IBM SPSS Inc., Chicago, IL). Questions with no response were treated as missing values in the analysis process. Participants who completed the first section of the questionnaire were included in the analysis even though some of them did not complete all the questions in the second section. Results were summarised using descriptive statistics and bivariate associations was assessed using Chi square test. Spearman correlation test was used to evaluate the correlation between categorical variables. For all statistical analysis, the significance level will be set at *p* < 0.05.

## Results

Responses were received from 252 dental practitioners in NSW, Australia. After removing the responses with missing data, 206 responses were included in the analysis. Participants in this study had an approximately equal gender distribution with the mean age of 47.5. Majority of the participants were general dentists from private sector. (Table 1). Majority of the participants (73.3%) have received their primary dental qualifications from Australia.

**Table 1:** Demographic characteristics of the participants

Characteristics		
Age (years)	Minimum	24
	Maximum	87
	Mean (SD)	47.5 (13.5)
Gender n (%)	Male	93 (45.1)
	Female	98 (47.6)
	Prefer not to answer	15 (7.3)
Practice sector n (%)^a^	Local health district	16 (7.8)
	Private practice	159 (77.2)
	Corporate dental provider	23 (11.2)
	University teaching clinic	9 (4.4)
	Australian defence forces	2 (1.0)
Practicing division ^b^	General dentist	180 (87.4)
	Specialist	24 (11.6)
	Oral health therapist/Dental therapist	2 (1.0)
Practice duration (years)	Minimum	1
	Maximum	54
	Mean (SD)	22.9 (14.0)
Practice frequency per week	More than 5 days per week	14 (6.8)
	5 days per week	66 (32.0)
	4 days per week	51 (24.8)
	3 days per week	29 (14.1)
	2 days per week	24 (11.7)
	1 day or less per week	7 (3.4)
	Not responded	15 (7.3)

^a^Some participants practice in multiple sectors

^b^There were no responses from dental hygienists or dental prosthetists

### Perception on COVID-19 infection risk in dental practice

**Fig. 2:**
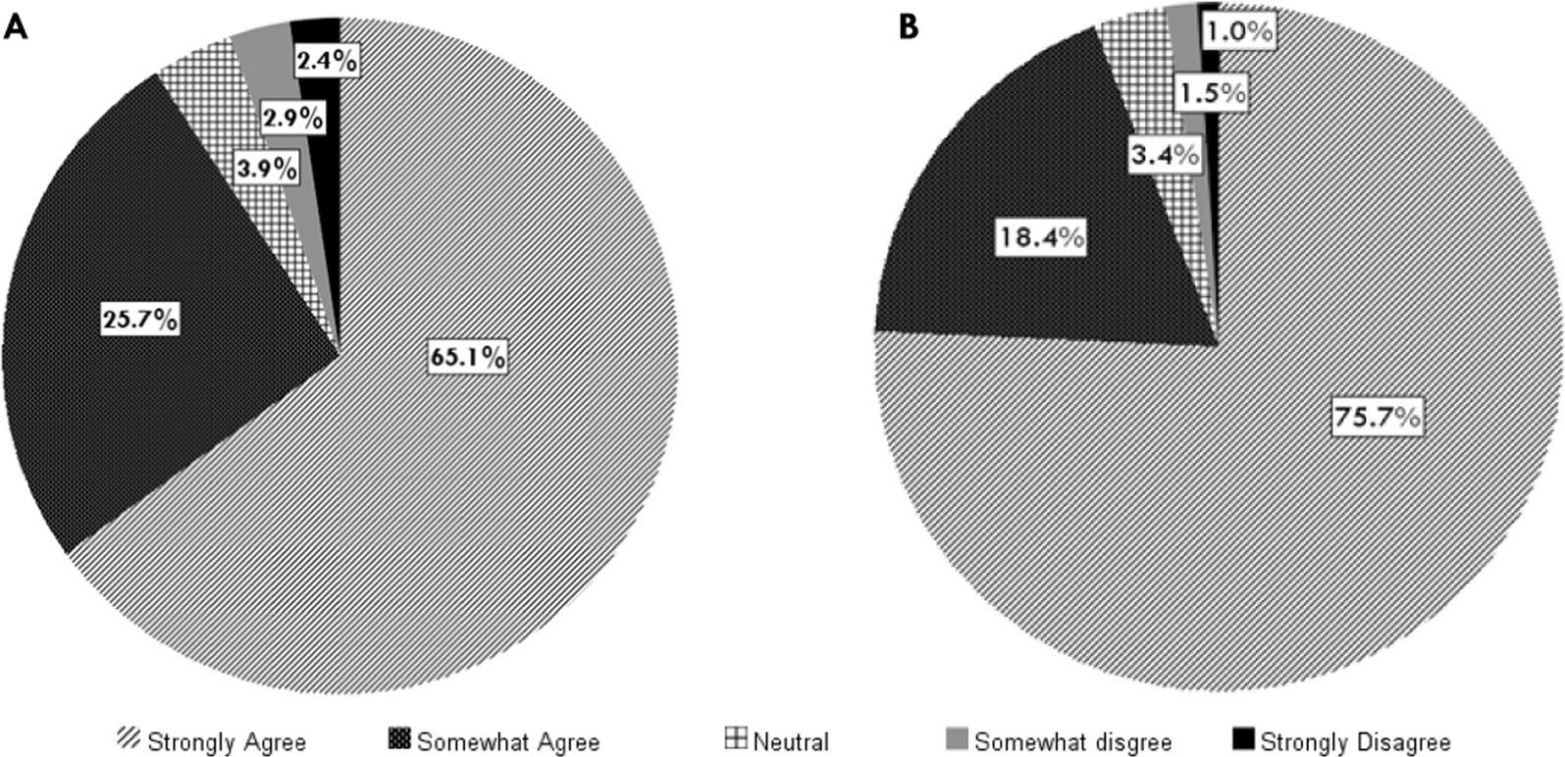
Attitude on risk of COVID-19 infection to dental practitioners. (**a**) Dental practitioners have a higher risk of infection with COVID-19 than many other health workers due to their professional work Aerosol generating procedures present a risk to dental practitioners being infected with COVID-19. (**b**) Aerosol generating procedures present a risk to dental practitioners being infected with COVID-19

As summarised in Fig. 2 over 90% of the participants agreed that dental practitioners have a higher risk of infection with COVID-19 than many other health workers due to the nature of their practice. The majority of the respondents reported that aerosol generating procedures in dental practice present a risk of COVID-19 infection to the dental practitioners (Fig. 2). It was also noted that 89.3% practitioners have deferred non-urgent dental treatment generating aerosols because of this risk and 81.6% of the practices have functioned only on an emergency basis during the level 2 and level 3 restrictions.

### Perception on patients’ accessibility to dental care

A majority of dental practitioners surveyed (80.1%) recognised that the pandemic limits patients’ accessibility to oral health care. Over 75% of the dental practitioners expressed concerns that patients will decide to defer dental treatment due to fear of COVID-19 infection. Interestingly, only about quarter of dental practitioners strongly believed that the negative impact of the pandemic on patients’ financial situation limited access to dental care (Table 2).

**Table 2:** Dental practitioners’ perception on patients’ accessibility to dental care

	Strongly agree	Somewhat agree	Neither agree nor disagree	Somewhat disagree	Strongly disagree
I am concerned that patients will decide to defer dental treatments because of fear of being infected by COVID-19 (n = 206)	80 (38.8)	76 (36.9)	23 (11.2)	20 (9.7)	7 (3.4)
I am concerned about the impacts of the COVID-19 pandemic on the Australian public’s ability to access oral healthcare (i.e., dental restrictions, limited bookings per day due to longer appointment for stricter infection control procedures) (n = 206)	80 (38.8)	85 (41.3)	26 (12.6)	11 (5.3)	4 (1.9)
I am concerned about the impacts of the COVID-19 pandemic on patients’ ability to fund their dental care (n = 206)	57 (27.7)	77 (37.4)	40 (19.4)	24 (11.7)	8 (3.9)

### Attitudes on guidelines provided by professional regulators

**Fig. 3:**
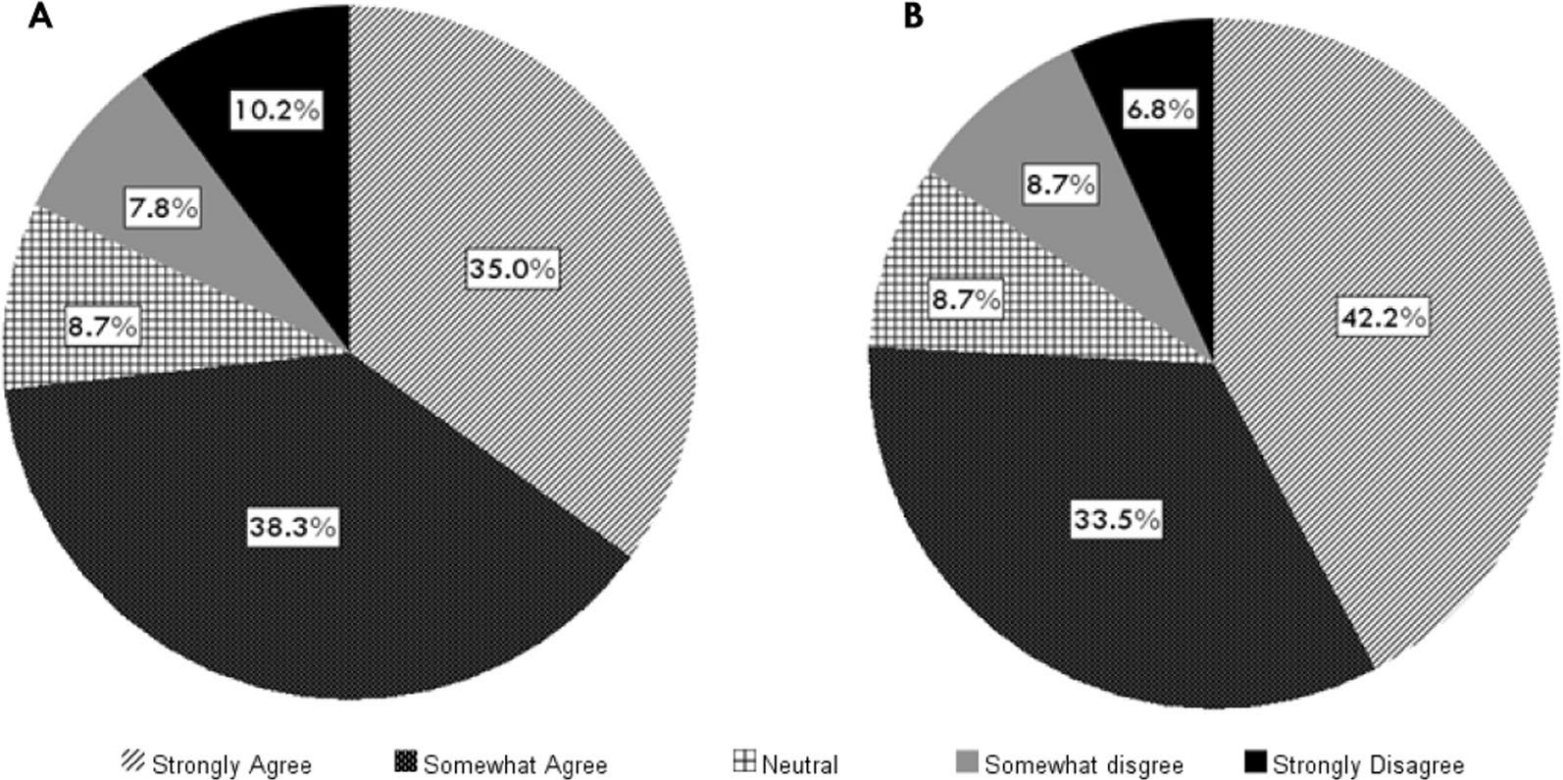
Attitudes of the participants on the guidelines provided by professional organisations and regulators. (**a**) I felt supported by the guidance I received from professional regulators on the COVID-19 pandemic to ensure the safe delivery of oral health care. (**b**) Guidance on restrictions to dental practice provided by the Australian Dental Association Inc. were helpful to my practice

Survey questions also explored the attitude of the participants on the level of guidance received from the professional regulators such as ADA Inc. The majority of the respondents either strongly agreed or agreed (73.3%) that they were supported by the guidelines received from professional regulators on COVID-19 pandemic to ensure the safe delivery of oral health care. Responses demonstrated that the guidelines from ADA Inc. are the most widely followed (80.1%) in their daily practice. About 70% participants also reported that they followed the government guidelines (NSW Health) at the same time. 75.7% of the participants reported that they felt supported by the guidance on restrictions to dental practice provided by ADA Inc. (Fig. 3).

### Measures adopted to deliver safe clinical care during the pandemic

#### Infection control measures of in-person dental care

**Fig. 4:**
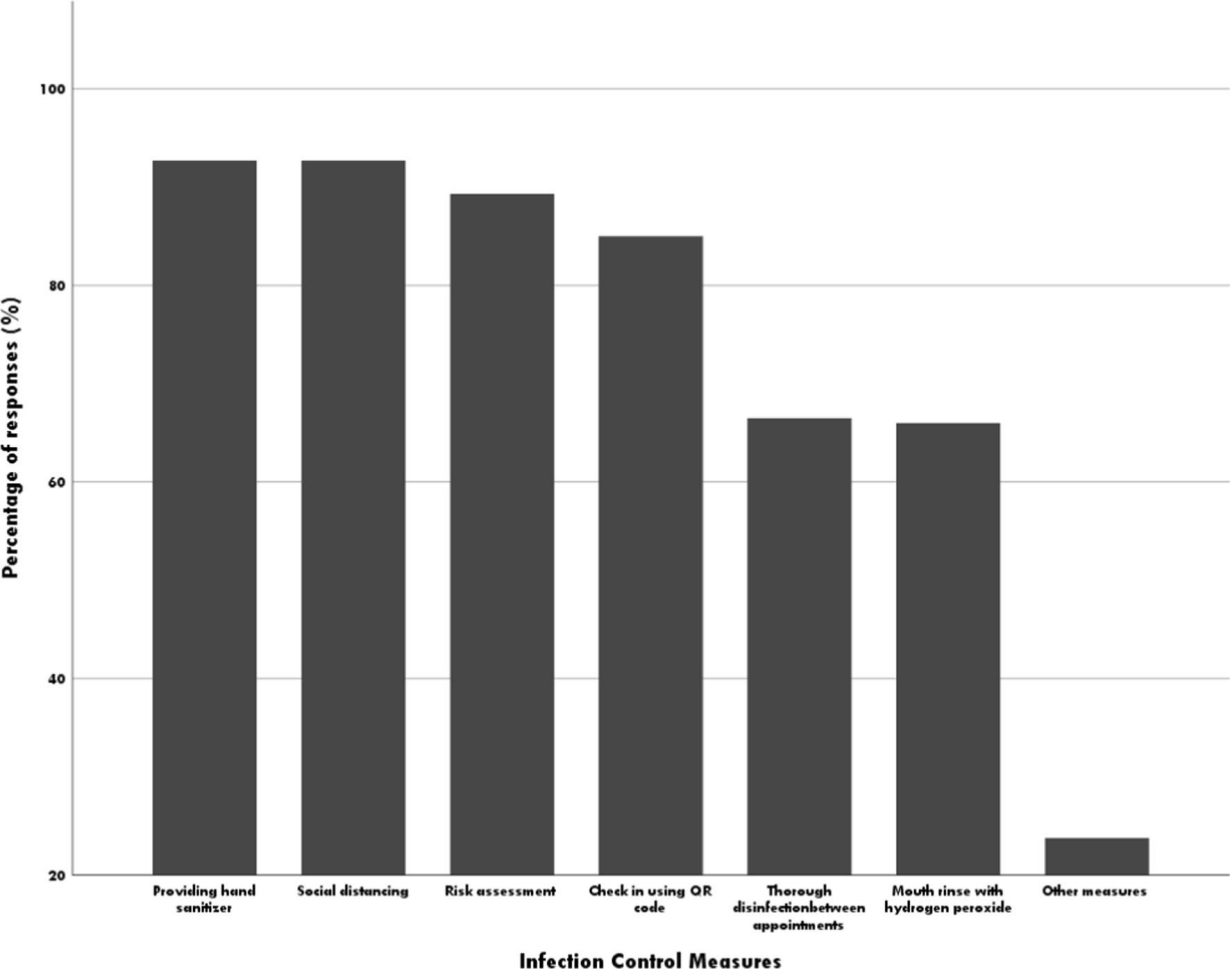
Infection control measures used in dental practices

The survey investigated the measures used to impede the transmission of the COVID-19 virus within the dental practices. Dental practitioners reported use of multiple infection control measures as shown in Fig. 4. Among those measures, providing hand sanitizer, social distancing, risk assessment and check-in using QR code were recognised as the most common measures. Over 60% of dental practitioners reported that using hydrogen peroxide mouth rinse before procedures. However, 5.8% of dental practitioners reported using other mouth rinse reagents such as Listerine and chlorhexidine. In addition, participants listed other infection control measures adopted in their patient care, including wearing face shields, minimizing aerosol generation procedures, requiring patients providing PCR test result or vaccination results, better ventilation of the waiting rooms, and requesting the patients to wait in their cars. Importantly, 60.7% dental practitioners mentioned that they preferred not to provide clinical care if the patients refuse to follow these measures while 21.4% would provide care anyway using appropriate PPE.

Participants were requested to select all infection control measures they use in their practices.

#### Patients’ perception on infection control measures on dental visit

Majority of the participants (63.9%) agreed that patients demonstrate a greater interest in infection control procedures. Almost all participants (96.4%) found that patients were considerate of social distancing requirements in the waiting room in dental practices following the COVID-19 pandemic (Table 3).

**Table 3:** Dental practitioners’ view on patients’ response to infection control measures

	Strongly agree	Somewhat agree	Neither agree nor disagree	Somewhat disagree	Strongly disagree
Patients openly demonstrate a greater interest in infection control procedures in dental practice following the COVID-19 pandemic (n = 205)	41 (20.0)	90 (43.9)	42 (20.4)	28 (13.7)	4 (2.0)
In practice, I find that patients are considerate of social distancing requirements in the waiting room (n = 196)	113 (57.6)	76 (38.8)	7 (3.6)	0	0

#### Tele-dentistry for remote dental care

Only 22.7% of the participants considered tele-dentistry as a useful alternative for providing patient care during service restrictions due to the pandemic. Almost half of the participants (45.9%) did not recognise tele-dentistry as a useful approach for patient care. Similarly, only 17.4% participants considered tele-dentistry in their practice even after the end of the current COVID-19 pandemic (Table 4).

**Table 4:** Dental practitioners’ view on tele-dentistry

Survey question	Strongly agree	Somewhat agree	Neither agree nor disagree	Somewhat disagree	Strongly disagree
Tele-dentistry services have been a useful resource for providing patient care in my practice during service restrictions (n = 194)	21 (10.8)	23 (11.9)	61 (31.4)	35 (18.0)	54 (27.9)
I feel that tele-dentistry will have a lasting place in my practice, even after the end of the current COVID-19 pandemic (n = 195)	15 (7.7)	19 (9.7)	50 (25.7)	48 (24.6)	63 (32.3)

### Perception on the impact on professional career

In this survey, over 60% of the dental practitioners reported that their clinics were closed for varying time durations causing financial hardships. This was evident with 59.7% and 21.4% of dental practitioners experiencing over 50% and 30–50% revenue loss respectively. However, there has been no additional fee from the patients due to cost of PPE in majority of these practices (76.7%). Over 40% of the participants were concerned that the pandemic may have long term financial impact to their practice.

Majority of participants (65.1%) reported they feel anxious about the potential future implications of the COVID-19 pandemic on their practice of dentistry such as practice safety and financial health. About 1 in 4 participants have considered changing their practice environment, moving sectors and even leaving their career in dentistry. However, majority of the dental practitioners reported they are willing to stay in their current practice environment and continue their career in dentistry. During the restriction period, even though only a limited number of practitioners (9.2%) have been re-deployed into another healthcare role, 60.2% expressed willingness to support to another area of the healthcare workforce, if the opportunity arises.

It is noteworthy that there was no statistically significant association between the responses for any of the Likert scale questions and the participant’s clinical qualifications (general practice vs specialised) or gender (Additional file 1: Table 1). Further, the dental practitioners’ responses were not significantly correlated with the duration of clinical experience (*p* > 0.05).

## Discussion

This survey was conducted to investigate the attitudes and perceptions of dental practitioners in NSW, Australia on the impact of the COVID-19 pandemic on the infection prevention and control practice, alternative dental service provision, financial impact, and career redeployment..

Restrictions were placed on dental services as a necessary course of action to reduce the transmission of virus and protect the public during the pandemic. Dental practitioners relied on the available guidelines such as infection prevention and control protocols recommended by the regulation and professional bodies to deliver dental care to patients. Common effective infection control measures such as providing hand sanitizer, social distancing, risk assessment and check in using QR code were widely adopted but not all dental practitioners applied all those measures in their daily practise. Apart from the universal precaution methods of using preventive barriers, there were not detailed guidelines for standard infection control procedures for dental patient care recommended by the professional bodies. The findings of this study have demonstrated different practice protocols on infection control measures were adopted based on individual dental clinic or dentist. A good example we observed that clinicians provided patients with different types of mouthwashes prior to the procedure to minimise the virus load in the oral cavity. The most widely applied mouthwash was hydrogen peroxide which has been proven safe and effective in reduction of SARS-Cov-2 virus load [19]. However, some dentist participants indicated the use of other mouthwashes such as Listerine mouthwash which lacks strong research evidence to support effectiveness in viral load reduction. This suggests that it is important for professional or regulation bodies to provide evidence-based clinical guidelines on the choice of infection control measures. Though a recent study showed dentists demonstrated sufficient knowledge and preparedness on COVID-19 [16], education programs and trainings such as continuing professional development would also be beneficial to dental practitioners to obtain updated knowledge and skills on infection control practice during the pandemic.

Dental practitioners are front-line health workers and have direct contact with patients during their routine clinical practice. Tele-health is advocated as one of the alternative measures to provide clinical care during the COVID-19 pandemic. Previous studies have reported that tele-dentistry is useful in providing distant dental screening, diagnosis, consultation, and treatment planning for patients during the pandemic to minimize non-essential physical interactions [20, 21]. However, most of the dental practitioners participated in this study neither recognised tele-dentistry as a useful alternative to provide patient care nor considered tele-dentistry in their practice after the pandemic. This finding conflicts with previous studies where high percentage of dentists considered tele-dentistry could be useful in their practice and the majority would even consider practicing it after the pandemic [22]. This variation indicates the impacts of geographical, technological, and attitudinal factors on the perception and acceptance of adopting tele-dentistry. In the Australian context, this may reflect the lack of financial remuneration available to support tele-dentistry from health insurers and the potential unpalatability for providers to charge patients directly for such care. This contrasts with the arrangement in general medical practice where telehealth consultations have been supported through the Commonwealth Government’s Medicare scheme. In the NSW public oral health service, tele-dentistry has been widely adopted and is recognised as contributing towards the management of waiting-lists through early identification of treatment needs and direction towards appropriate care pathways.

Dentists are integral members of the healthcare workforce during the pandemic. Equipped with knowledge and skills on patient management, our study showed that most dentists were willing to be redeployed and reutilised during the COVID-19 pandemic to contribute to efforts to manage the pandemic. Sadly, due to dentistry’s exclusion from much of the wider health system, few dental professionals based within the private sector were able to contribute their skills externally to the pandemic response [23]. In contrast, dental practitioners from the public oral health services were widely redeployed during practice restrictions, engaging in activities such as swabbing and vaccination preparation and administration. Undoubtedly, the COVID-19 pandemic has caused profound financial ramifications for the whole community including dental practices. The financial impacts on dental practices during the pandemic due to reductions in patient volume and additional costs for infection control measures have been well-documented globally [21, 24, 25]. In addition, this study shown most dental practitioners were concerned on their own health wellbeing and infection risk that could also lead to the reduction of clinical practice hours and consequently affect the financial income. It is important to note that while the pandemic saw prices and demand for PPE skyrocket, respondents did not report passing the cost of this onto patients.

At the time of writing, dentistry in Australia is cautiously recovering from the period of instability that saw practice restrictions and increased infection control procedures. Despite this welcome recovery, this research demonstrates that greater alignment and incorporation of dental practice within the wider Australian health system would assist in dental professionals being able to contribute their skills more broadly to the promotion of health, both during times of routine and in crisis.

## Limitations

Some limitations of this study need to be stated. Even though the survey invitation was sent to all the dental practitioners registered in NSW Australia, due to the voluntary nature of the participation, the study may not have captured the responses from a representative sample of the target population. Further, not being able to achieve the expected sample size is another limitation and may have skewed the results. There was limited participation from dental specialists, dental hygienists, therapists, oral health therapists, and dental prosthetists. Due to the cross-section design, this study will not reflect the observations across different phases and stages of the pandemic.

## Conclusion

The results of our study demonstrate the systematic disruption to dental practice faced in Australia and challenges experience by the dental practitioners due to the COVID-19 pandemic. While the survey responses demonstrate positive findings relating to levels of support that respondents felt from professional associations, the value of evidence-based clinical guidelines on clinical practice such as the choice of infection control measures needs to be highlighted. The pandemic has also exposed the disconnection of the dental care system from the rest of healthcare. To improve access to dental care services both during times of routine and crisis, adoption of technologically driven models of care which have been widely utilized in other health sectors should also be supported and promoted in dentistry.

## Supplementary Information

**Additional file 1: Supplementary Table 1.** Bivariate analysis results.

## Data Availability

The dataset used and analysed during the current study is available from the corresponding author on reasonable request.
