# Supplementary material for: Perceptions and attitudes of dental practitioners towards impacts of Covid 19 pandemic on clinical dentistry: a cross-sectional study
Source: BMC Oral Health. 2022 Sep 22;22:424. doi: 10.1186/s12903-022-02457-y (PMC9502939; doi:10.1186/s12903-022-02457-y)
Supplement: Supplementary file 1 — Additional file 1: Supplementary Table 1. Bivariate analysis results. [file 12903_2022_2457_MOESM1_ESM.docx]

|  | Gender | | | Clinical Qualification | |
| --- | --- | --- | --- | --- | --- |
|  | Male | Female | Prefer not to answer | General dental practitioners | Dental specialist |
| Q: I feel anxious about the future implications of the COVID-19 pandemic on my practice of dentistry (i.e., in practicing safety or financially). | | | | | |
| Strongly disagree | 7 | 7 | 1 | 14 | 0 |
| Somewhat disagree | 8 | 12 | 0 | 18 | 2 |
| Neither agree nor disagree | 17 | 17 | 3 | 31 | 4 |
| Somewhat agree | 35 | 36 | 6 | 57 | 12 |
| Strongly agree | 26 | 26 | 5 | 49 | 6 |
|  | χ2 =2.56, df=8, p>0.05 | | | χ2 =3.80, df=4, p>0.05 | |
|  |  |  |  |  |  |
| Q: The COVID-19 pandemic has led me to consider leaving my career in dentistry. | | | | | |
| Strongly disagree | 38 | 53 | 6 | 83 | 10 |
| Somewhat disagree | 16 | 16 | 2 | 25 | 5 |
| Neither agree nor disagree | 13 | 5 | 4 | 15 | 4 |
| Somewhat agree | 15 | 18 | 3 | 29 | 5 |
| Strongly agree | 11 | 5 | 0 | 16 | 0 |
|  | χ2 =13.67, df=8, p>0.05 | | | χ2 =4.44, df=4, p>0.05 | |
| Q: The COVID-19 pandemic has led me to consider changing the environment in which I practice dentistry (i.e., moving sectors or practice type) | | | | | |
| Strongly disagree | 38 | 40 | 6 | 70 | 9 |
| Somewhat disagree | 15 | 16 | 0 | 24 | 5 |
| Neither agree nor disagree | 16 | 16 | 4 | 30 | 3 |
| Somewhat agree | 17 | 17 | 4 | 31 | 5 |
| Strongly agree | 7 | 8 | 1 | 13 | 2 |
|  | χ2 =3.89, df=8, p>0.05 | | | χ2 =1.11, df=4, p>0.05 | |

Supplementary Table 1. Bivariate analysis results
